# Home-based exercise interventions’ impact on breast cancer survivors’ functional performance: a systematic review

**DOI:** 10.1007/s11764-024-01545-y

**Published:** 2024-02-15

**Authors:** Pedro G.F. Ramos, Pedro B. Júdice, Inês Nobre, Eliana V. Carraça

**Affiliations:** 1 Universidade Lusófona, Campo Grande 376, Lisboa, 1749-024 Portugal; 2 Centro de Investigação em Educação Física, Exercício e Saúde (CIDEFES), Universidade Lusófona, Campo Grande 376, Desporto, Lisboa, 1749-024 Portugal; 3 Centro Interdisciplinar de Performance Humana (CIPER), Faculdade de Motricidade Humana, Estrada Costa Cruz Quebrada, Cruz Quebrada-Dafundo, 1495-688 Portugal

## Abstract

**Introduction:**

Home-based exercise (HBE) programs can be a feasible strategy to enhance functional performance and promote physical activity (PA) in breast cancer survivors. A deeper analysis of the effects of HBE interventions, structured by HBE program type and treatment phase, is needed. This systematic review aimed to synthesize the evidence on HBE interventions’ impact on breast cancer survivors’ functional performance, PA levels, and program adherence rates, according to HBE intervention type and treatment phase.

**Methods:**

A comprehensive search of peer-reviewed articles reporting HBE interventions’ effects on the outcomes of interest was performed in Pubmed, Google Scholar, EBSCO, Web of Science, Science Direct, and B-ON until January 15th, 2024. Data were synthesized according to Denton’s domains to classify HBE interventions (prescription: structured vs. unstructured; Delivery method: supervised vs. facilitated vs. unsupervised) and treatment phase. Methodological quality appraisal was performed using the Effective Public Health Practice Project tool.

**Results:**

Twenty-six studies were included. Most studies conducted structured/facilitated interventions and reported positive effects on functional performance (particularly aerobic capacity), increases in PA levels, and high adherence rates (> 70%) during and post-treatment.

**Conclusion:**

HBE interventions may be feasible to improve functional performance and promote physical activity among breast cancer survivors. Further studies are needed to confirm which HBE intervention type is more appropriate for each treatment phase. More evidence applying HBE interventions with different designs is required to allow the drawing of more solid conclusions. Studies exploring the effects of HBE interventions on the pre-treatment phase are needed.

**Supplementary Information:**

The online version contains supplementary material available at 10.1007/s11764-024-01545-y.

## Introduction

Breast cancer is the most common type of cancer in women, having an 11.7% prevalence and causing 6.9% of deaths worldwide [1, 2]. According to recent data, breast cancer continues to have a significant impact worldwide, highlighting the need for health measures targeting cancer control across the disease continuum to mitigate breast cancer mortality and address the overall impact of this disease [3].

Although breast cancer treatments have been evolving and contributing to an increase in cancer survivorship, they carry several side effects that reduce survivors’ functional performance, affecting their independence and quality of life (e.g., cancer-related cachexia, cardiotoxicity, pain, and fatigue) [4–6].

Recent reviews have underscored the benefits of exercise in cancer, attesting their advantage at all stages of disease and treatment [7, 8]. Exercise and physical activity are potential strategies to reduce breast cancer’s risk of incidence, prevalence, recurrence, and cancer-related mortality [7, 9]. It can also contribute to a better cancer prognosis, potentiate treatment efficacy, manage disease and treatment-related side effects (e.g., cachexia, fatigue, depression, cardiotoxicity, arthralgia), and improve quality of life, and increase disease-free survival [7, 8]. Prior systematic reviews have also showed that exercise positively impacts aerobic capacity, strength, body composition, and quality of life in cancer patients undergoing neoadjuvant treatment [10], grip strength and VO_2_peak during adjuvant treatment (radiotherapy, chemotherapy) [11], and aerobic capacity and pain reduction in breast cancer survivors’ ongoing hormonal therapy [12].

Despite the known benefits, many survivors do not meet PA recommendations, underlining the importance of creating interventions that facilitate the adoption and maintenance of an active lifestyle [12–14].

Home-based exercise (HBE) programs (i.e., any program undertaken inside or in the immediate surroundings of one’s home) can raise adherence to PA since they are convenient and offer flexible scheduling [15, 16]. According to Denton’s categorization, HBE interventions can be structured (i.e., prescribed based on training principles and tailored to specific goals) or unstructured (i.e., activities may include daily tasks, not explicitly prescribed by a professional) [16]. It can be directly supervised (either virtually or in-person), facilitated (with no direct supervision but with scheduled professional consultations to monitor progress and give support), or unsupervised (with no professional presence during the practice or scheduled appointments) [16].

Prior systematic reviews have shown the effectiveness of HBE interventions in improving breast cancer survivors’ functional performance and PA levels [17–22]. However, none of these reviews seems to have investigated the impact of different HBE interventions in cancer survivors (i.e., from diagnosis to the end of life) throughout the various phases of treatment, analysing and organizing the results according to Denton’s categorization [16].

As a result, this systematic review aimed to synthesize the evidence on adherence rates and effects of HBE interventions on breast cancer survivors’ functional performance and PA levels, according to the type of HBE intervention and treatment phase.

## Methods

This review followed the Preferred Reporting Items for Systematic Reviews and Meta-Analyses (PRISMA) guidelines, and was registered on PROSPERO after the search was completed (CRD42023411803) [23].

### Eligibility criteria

Included studies were randomized controlled trials (RCT), published in English until January 15th, 2024 (including online ahead of print publications), involving stage 0-III breast cancer diagnosis, reporting differences between one or more HBE intervention groups and a control group. These studies had to measure at least one of the following outcomes: functional performance and PA levels or rates of adherence to the program.

Studies involving other types of cancer, or a stage IV diagnosis were excluded since these stages require supervised care and are usually found in palliative care. Interventions that combined other disciplines (e.g., nutrition) or presential sessions as part of the program were also excluded to allow isolation of HBE intervention’s effects.

### Search strategy

A comprehensive search for peer-reviewed articles published until January 15th, 2024 (including online ahead of print publications) was performed in various databases (Pubmed, Web of Science, Science Direct, Google Scholar, EBSCO, and B-ON). No starting date was defined as a criterion. Additionally, manual searches were performed on Pubmed and Google Scholar, and bibliographies were cross-checked. Authors were contacted to provide access to articles in case of unavailability.

The search strategy followed PICOS and included five sets of keywords and respective derivatives, synonyms, and combinations: P: adult, breast cancer survivors/patients; I: home-based physical activity/exercise; O: adherence, physical activity, functional performance; S: Randomised Controlled Trial.

Two independent authors (PGFR, IN) performed the searches and used CADIMA to screen and select articles by title and abstract [24]. After the screening, the two authors performed a consistency test on CADIMA to ensure the application of the same criteria in the articles’ appraisal and selection. The potentially eligible articles were retrieved and divided among the two authors (PGFR, IN) for examination. Doubts were discussed with a third author (EVC or PBJ) and resolved by consensus.

### Data extraction and coding

Two authors (PGFR, IN) performed data extraction. The extracted data included study details (authors, year, country), sample characteristics, phase of treatment, HBE type, intervention design and brief description, outcomes of interest (instruments), and main findings. Any divergences or doubts were solved through discussion and analysis with the other two authors (EVC, PBJ).

### Outcome measures

Functional performance refers to a person’s capability to complete daily activities involving physical effort [25]. It was evaluated by considering aerobic capacity (walking tests and VO_2_ measures), muscle strength (1-repetition maximum and multiple repetition tests, handgrip, sit-to-stand, arms lifting, muscle testing techniques), and range of motion.

PA was measured by considering the volume (daily or weekly minutes of activity, self-reported or objectively measured), intensity (light, moderate, vigorous), and type (walking, resistance, and others).

Program adherence was defined as the participant’s level of attendance or completion of the exercise sessions and its prescription [26]. Program adherence was assessed through participants’ self-reported information (i.e., questionnaires and exercise logs) or objectively collected data (accelerometer, activity trackers, and pedometer).

### Quality assessment

The Effective Public Health Practice Project (EPHPP) tool assessed the studies’ risk of bias [27]. This tool was used based on several authors’ recommendations [27–29] and shows appropriate content and construct validity [26–30]. It appraises the methodological quality of the studies according to eight domains: study design, blinding, selection bias, withdrawals/dropouts, confounders, data collection, data analysis, and reporting. Each domain is classified as strong, moderate, or weak based on specific criteria, and their combined score provides an overall quality rating of the evidence (i.e., weak if two or more domains are classified as weak; moderate if one domain is classified as weak, and strong if all the domains are classified as moderate or strong). Two authors (PGFR, IN) critically appraised all the articles selected, and any disagreements were solved through consensus or discussion with a third author (EVC, PBJ).

### Data synthesis

A narrative synthesis of the evidence on the effects of HBE interventions on functional performance, PA levels, and program adherence rates was conducted and presented in tabular form. The information was organized by type of HBE intervention (structured/unstructured; supervised/facilitated/unsupervised) and cancer treatment phase (pre-, during, or post-treatment).

The World Cancer Research Fund (WCRF) and the Institute of Medicine define a cancer survivor as someone living with the disease, regardless of its stage and treatment phase, from diagnosis until death [29–32]. The cancer survivorship continuum comprises three phases: pre-treatment, during treatment, and post-treatment [33].

HBE intervention types were classified based on the domains proposed by Denton et al. [16]. Accordingly, HBE programs were classified as unstructured when no prescription was delivered by a healthcare/exercise professional or structured if the program was prescribed based on the Frequency, Intensity, Time, and Type (FIIT) principles and with a specific health or fitness goal; unsupervised, in interventions performed without the presence, support or progress tracking from a qualified professional, facilitated when the activities were performed without supervision, but had professional counselling and monitoring along the program, or supervised if the activities occurred virtually led by an exercise professional to ensure safety and correct technique instructions.

Cancer treatment phases were categorized into pre-treatment, during treatment, and post-treatment, accounting for the participants’ active treatment phase (surgery, chemotherapy, and radiotherapy) [29, 34].

## Results

### Literature search

**Fig. 1:**
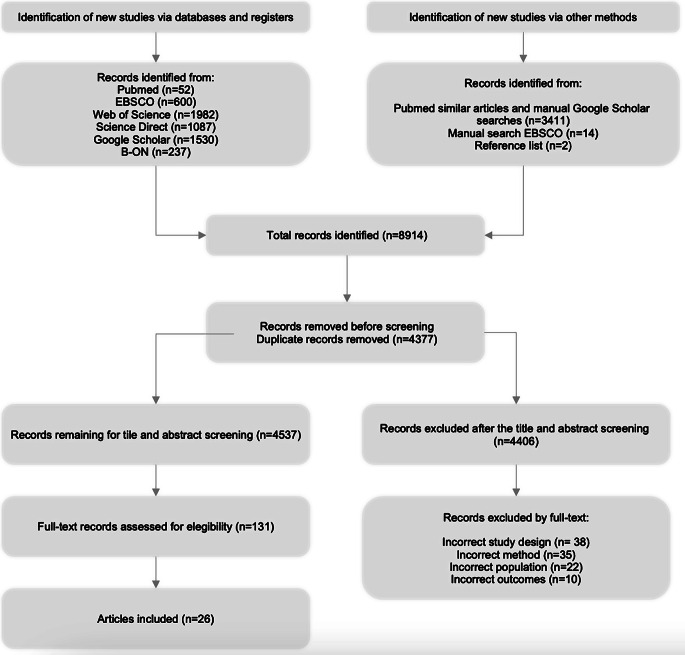
PRISMA flow diagram

A total of 8910 records were added to CADIMA after all database and manual searches were concluded. After duplicate removal, 4533 were screened by title and abstract, and 127 articles remained for full-text screening. Twenty-six articles were included in this review. The PRISMA flowchart is presented in Fig. 1.

### Study characteristics

Descriptive data for the studies included in this systematic review (*n* = 26) are summarised in Table 1.

**Table 1:** Studies design, sample, intervention, and outcomes characteristics

Study characteristic	N	Study characteristic	N
**Design characteristics**		**Equipment(s) used in intervention**	
2-arm RCT^1^	23	No equipment	19
3-arm RCT	3	No equipment (aerobic) + free weights^d^ and/or elastic bands (resistance)	3
**Sample**		No equipment or cycle ergometer (aerobic)	1
**Sample size**		Not reported/specified	3
< 25	2	**Self-monitoring tools**	
25–49	7	Self-report log + self-monitoring instructions	4
50–74	7	Wearables^c^	5
75–99	6	RPE^4^ scale	1
>=100	4	Symptoms screening	1
**Age range (years)**		Self-report logs + RPE scale and/or wearables	15
≥ 18^a^	1	**Delivery mode**	
45-54.9	21	Phone calls	14
55–64	3	Apps and email	1
≥ 65	1	Televideo	1
≥ 65	1	Combined modes^b^	8
**Treatment Stage**		Unsupervised	2
Pre-treatment	0	**Control group**	
During treatment	13	Usual PA^5^ levels/care/ Waiting list	12
Post-treatment	13	Could do PA but no access to study’s program	1
		Hospital’s usual treatment/care	3
**Intervention**		Educational phone calls	1
**Intervention Length**		Usual PA levels + follow-up phone calls	4
< 4 weeks	1	Informational materials	1
4–12 weeks	15	Standard PA information	1
13–24 weeks	6	No details on the condition	1
> 24 weeks	2	Usual PA levels/care + follow-up phone calls + informational materials	1
Adapted duration^2^	2	Waiting list + wearables	1
**Home-based exercise program**		**Quality analysis (EPHPP)**	
Structured; facilitated	19	Weak	21
Structured; supervised	1	Moderate	5
Structured; unsupervised	2	**Outcomes**	
Unstructured; facilitated	4	**Functional Performance**	**17**
**Intervention program**		Aerobic capacity	9
Aerobic training	12	Aerobic capacity + strength	4
HIIT	1	Aerobic + self-reported	3
Aerobic or resistance training	2	Range of motion + strength	1
Combined aerobic + resistance	5	**PA Levels**	**17**
Mobility	1	Self-reported logs	12
Aerobic + balance + stretching	1	Objectively measured (wearables)	1
Unstructured program	4	Logs + questionnaire	2
**Intervention delivery**		Wearable + Logs	1
Initial instruction (no material delivered)	9	Questionnaire + wearables	1
Personal/ Phone meetings + written materials /video	7	**Adherence**	**15**
Delivery details not reported	1	Wearables	3
Face-to-face televideo sessions	1	Self-reported logs	8
Written materials	2	Presence registration	1
Exercise videos	1	Logs + wearables	3
Written materials + video	2		
No intervention delivery details	2		
Email + smartphone app	1		

Abbreviations: ^1^ RCT, Randomized Controlled Trial; ^2^Adapted duration, 6-week radiotherapy or 3–6 months chemotherapy; ^3^HIIT, High-Intensity Interval Training; ^4^RPE, Borg’s Rating of Perceived Exertion Scale;, ^5^PA, Physical Activity

^a^Participants’ age was reported in range (no mean age available)

^b^Phone calls and/or in-person consultations/ meetings and/or emails

^c^Wearables used included: heart rate monitors, pedometers, smartwatches and/or activity trackers

^d^Free weights included: multi-weight dumbells, Gymstick

The studies included in this review were published between 2001 and 2023 and reported RCTs comparing one HBE intervention group (*n* = 21) [35–57] or two HBE intervention groups (*n* = 3) against a control group [58–60].

Sample. Data from 1680 women between 27 and 78 years old diagnosed with stage I-III breast cancer were analyzed. Sample sizes ranged from 15 to 159. Most studies were carried out during treatment (*n* = 13) [36, 37, 39, 42–44, 48, 49, 53–56, 58] and post-treatment (*n* = 13) [35, 38, 40, 41, 45–47, 50–52, 57, 59, 60]. During treatment, interventions were carried out post-surgery (*n* = 2) [44, 61], during neoadjuvant treatment (*n* = 1) [53], adjuvant therapy (*n* = 9) [36, 39, 42, 43, 48, 49, 55, 56, 58] and both (*n* = 1) [37]. Post-treatment interventions ranged from four months to five years after treatment completion [35, 38, 41, 45–47, 50–52, 57, 59, 60].

Intervention. The interventions’ length ranged from 11 days to 8 months. Eighteen studies included structured and facilitated HBE interventions [35, 37, 39, 42, 43, 47–59] one structured and supervised [38], two structured and unsupervised [40, 44] and five unstructured and facilitated programs [36, 41, 45, 46, 60]. Thirteen interventions consisted of walking programs [35, 40, 42, 47–49, 51, 52, 54, 55] walking and other aerobic activities [53], walking or other self-chosen aerobic activity [58–60] performed at moderate to vigorous intensity, with variable frequency (from 2 to 3 to 5–7 days) and duration (from 20 to 50 min/session).

Three programs focused solely on resistance training [50, 58, 59] comprising exercises for the upper and lower body, using bodyweight [50], resistance bands [61], and dumbbells [59], performed 3–4 times per week, using several methodologies, including high-intensity interval training delivered through a smartphone app [50].

Five studies encompassed aerobic and resistance interventions [37–39, 43, 56] involving walking [39, 43] other aerobic activities [37, 38] and upper and lower body strengthening exercises (1–3 times/week) [37, 43]. One study included a shoulder mobility and flexibility post-mastectomy HBE rehabilitation program [44]. Four studies delivered unstructured programs consisting of generic aerobic exercise guidelines to be performed through self-chosen activities [36, 41, 45, 46]. One study conducted an intervention comprising walking, balance exercises, and stretches [57].

Delivery modes included in-person instruction sessions or printed materials, alone or combined with audio-visual materials [35–37, 39–42, 44–46, 48–54, 56–59]. One supervised intervention was delivered through televideo and phone apps [38]. In facilitated interventions, participants’ activities were mainly delivered through phone calls alone [35–37, 39, 42, 43, 47, 49, 51, 53, 55, 57–59] or combined with in-person meetings [48, 54] or emails [41, 45, 50, 52, 60].

No adverse effects were reported during nine HBE interventions [36–38, 45, 49, 50, 53, 55, 59]. Adverse effects related to exercise were reported in some studies, such as anemia, dizziness, and dyspnoea [54], shoulder pain and swelling of the axillary incision [44], knee discomfort [43], muscle soreness [39, 56], muscle injury [39], and sprained ankle [56].

### Outcomes

Seventeen studies assessed and reported effects on functional performance [37–39, 43–45, 48–54, 57–60]. These studies tested aerobic capacity (*n* = 8) [43, 51–53, 57, 59–61] strength (*n* = 1) [44], aerobic capacity and strength (*n* = 4) [37, 38, 50, 58], self-reported functional performance (*n* = 1) [39], self-reported functional capacity and aerobic capacity (*n* = 2) [48, 49], and shoulder mobility and strength (*n* = 1) [44]. Aerobic capacity was measured with walking, treadmill or cycle ergometer tests (*n* = 8) [43, 48–52, 54, 57, 59], direct maximal oxygen uptake (VO_2peak_) measurement (*n* = 1) [38], or using a combination of both tools (i.e., test and gas exchange analysis) (*n* = 5) [37, 46, 50, 53, 60]. Strength was assessed through a maximum repetition test (*n* = 1) [58], functional task performance (*n* = 1) [38], grip strength (*n* = 1) [44], both maximum repetition, functional task performance and grip strength (*n* = 1) [50], isometric bench test (*n* = 1) [37], and muscle testing techniques (*n* = 1) [44]. One study measured the shoulder range of motion using standard goniometric techniques [44]. Functional performance was assessed in structured/facilitated (*n* = 12), structured/unsupervised (*n* = 1), structured/supervised (*n* = 1), and unstructured/facilitated (*n* = 1) HBE programs, taking place during (*n* = 8) [37, 43, 44, 48, 49, 53, 54, 58] or post-treatment (*n* = 7) [38, 46, 50–52, 59, 60].

Seventeen studies investigated changes in participants’ PA levels of the participants, analyzing data from questionnaires (*n* = 10) [35, 39, 43, 45, 46, 52–56], exercise diaries (*n* = 1) [48], accelerometer (*n* = 1) [41], or using a combination of methods (*n* = 6) [40, 47, 49, 51, 60]. PA assessments were performed in structured/facilitated (*n* = 13), structured/unsupervised (*n* = 1), and unstructured/facilitated (*n* = 2) HBE interventions during treatment (*n* = 7) [39, 43, 48, 49, 53–55] or in the post-treatment (*n* = 9) [35, 40, 41, 45–47, 51, 52, 60] phases.

Fourteen studies assessed and reported adherence to the program by analyzing activity diaries data (*n* = 9) [35, 40, 42–44, 47, 48, 57, 59], activity monitors (*n* = 3) [50, 53, 55], or both (*n* = 2) [37, 42]. Adherence was analyzed in structured/facilitated (*n* = 10), structured/unsupervised (*n* = 2), and unstructured/facilitated (*n* = 1) HBE programs during the treatment (*n* = 8) [36, 37, 42–44, 49, 53, 55] and post-treatment (*n* = 5) [35, 40, 47, 50, 59] phases.

### Quality assessment

From the included studies, five were classified as moderate [38, 39, 42, 44, 50], while most were classified as weak (*n* = 19) [35–37, 40, 41, 43, 45–49, 51–60]. No studies reported on participants’ blinding until recruitment was completed. Only two studies reported having controlled for confounding factors such as length and type of treatment. All participants were selected from a convenience sample from hospitals, oncology centres, or clinics. Detailed information on quality assessment can be found in the supplementary Table 1 (online version).

## Main results

The main results of this systematic review are summarised in Table 2. More detailed information can be found in the supplementary Table 2 (online version). It should be noted that no studies reporting the pre-treatment phase were found. Also, no unstructured and unsupervised HBE interventions were found in the included studies.

**Table 2:** Home-based intervention outcomes categorised by type and treatment phase

Treatment Stage (n)	Adherence (n)	Physical activity (n)	Functional performance (n)
**Structured facilitated**			
During treatment (*n* = 11)	72–96% (7) N/R (4)	**Self-reported** ↑(4) ↔(4) N/R (3)	**Aerobic capacity**: ↑(4) ↔ (2) ↑(1) aerobic group/ ↔resistance group N/R (3) **Strength**: ↔(1) ↑(1) aerobic group/↔resistance group N/R (8) **Self-reported** ↑(3) N/R (6)
Post-treatment (*n* = 8)	73–90% (5) N/R (3)	**Self-reported** ↑(5) **Objectively measured** ↔(1) ↑(1) low-intensity group/ ↔high-intensity group (1) ↑pedometer steps/ ↔accelerometer N/R (2)	**Aerobic capacity** ↑(4) ↔(1) ↑(1)VO_2peak_/↔6MWT ↑(1)resistance group/ ↔aerobic group N/R (1) **Strength** ↑(1) leg press/↔Grip strength/ ↔Chair stand test N/R (5)
**Structured/ supervised**			
Post-treatment (*n* = 1)	N/R	N/R	**Aerobic capacity**↔ **Strength**↑
**Structured; unsupervised**			
During treatment (*n* = 1)	50%	N/R	**Shoulder ROM** (flexion and abduction)↑ Shoulder external rotation↔ **Strength**↔
Post-treatment (*n* = 1)	80%	Self-reported↑	N/R
**Unstructured; facilitated**			
During treatment (*n* = 1)	90%	N/R	N/R
Post-treatment (*n* = 3)	N/R (4)	Self-reported ↑(2) Objectively measured ↑(1)	**Aerobic capacity** ↑(1) N/R (2)

**Abbreviations**: ↑, positive effect; ↔ no effect; ↓ negative effect, in comparison with usual care group; n, number of studies; N/R, not reported variable/ information unclear/ assessment to variable was not performed; ROM, Range of Motion

### Structured and unsupervised HBE interventions

One study included a structured and unsupervised HBE intervention during treatment [47], reporting positive effects on shoulder flexion and abduction range of motion but no effects on strength. Grip strength increased in both groups, with no differences between them. Improvements in external shoulder rotation were found in both groups, with results favouring the control group. This study reported a 50% adherence.

One study included a structured and unsupervised intervention in the post-treatment phase [39], reporting an 80% adherence rate to the program and positive effects on PA levels.

Structured and facilitated HBE interventions.

Eleven studies included structured and facilitated HBE interventions during treatment [37, 39, 42, 43, 48, 49, 53–56, 58]. Four studies reported positive results in aerobic capacity [48, 49, 53, 54], and two showed no differences compared to usual care [37, 43]. Three studies showed positive effects on self-reported functional performance [39, 48, 49]. One study found no differences in strength between intervention and usual care [37]. Another study reported differences in aerobic capacity and strength in the aerobic group but not in the resistance group [58]. Out of the eight studies that assessed PA levels, six reported significant increases post-intervention [39, 48, 49, 53–55], and one showed no differences compared to usual care [43]. The mean adherence to the program ranged from 72 to 96% [37, 39, 42, 43, 49, 53, 55]. One study reported marginally better adherence to the program from participants undergoing chemotherapy (75%) than radiotherapy (72%) [49]. One study reported increases in PA levels in the intervention group participants but no significant differences between groups [56].

Five studies included this type of HBE intervention in the post-treatment phase. All studies reported positive effects on the participants’ aerobic capacity [50–52, 57], 59]. One study found differences in the resistance training but not in the aerobic exercise group [62]. Another study found no differences between groups [60]. One study assessed aerobic capacity, reporting positive effects on VO_2peak_ but not in the 6-minute walk test, and strength showing improvements in the 1-repetition maximum test but not in the chair stand test, compared to the control group [50]. All studies reported positive effects on participants’ self-reported PA, but the same was not confirmed for objectively measured PA [35, 47, 51, 52]. Program adherence levels ranged from 76 to 94% [35, 47, 50, 59]. One study reported a marginally higher adherence from the resistance training group than the aerobic exercise group (80% vs. 76%) [59].

### Structured and supervised HBE interventions

One study conducted a structured and supervised HBE intervention [38]. This intervention was conducted post-treatment, reporting positive effects in self-reported physical functioning and strength levels but no differences in aerobic capacity. The program retention rate was 87%.

### Unstructured and facilitated HBE interventions

One study applied a unstructured and facilitated HBE program during the treatment phase [36]. This study reported a 90% adherence to the program. No other relevant outcomes were reported in the article.

Three studies used unstructured and facilitated HBE programs in the post-treatment phase [41, 45, 46]. One study assessed functional performance, reporting potential benefits on aerobic capacity [46]. All studies reported increases in PA levels [41, 45, 46].

## Discussion

This systematic review is the first to synthesize the evidence on adherence rates and the effects of HBE interventions on breast cancer survivors’ functional performance and PA levels, according to the type of HBE intervention and treatment phase.

Most HBE interventions were structured and facilitated, showing significant effects on functional performance (particularly on aerobic capacity) and PA levels and very good adherence rates, both during the treatment and post-treatment phases. Unstructured and facilitated HBE interventions were the second most used type, mainly conducted post-treatment and presenting positive effects on functional performance (aerobic capacity) and PA levels. Structured/supervised and structured/unsupervised HBE interventions revealed good adherence (up to 80%), but remain poorly studied to allow solid conclusions regarding their effectiveness.

The dominance of aerobic-focused programs might explain the commonly reported improvements in aerobic capacity. Still, the findings were inconsistent across different types of training (i.e., aerobic, resistance, or combined) and assessment methods (i.e., walking tests, gas exchange measures), precluding the withdrawal of conclusions about which aerobic capacity component is the most improved in different types of HBE interventions and within different phases. Further studies are needed to shed some light on this issue.

One possible reason for the lack of consistent improvement in strength levels in response to HBE interventions might be related to the complexity of the techniques required in resistance exercises compared to tasks such as walking or other naturalistic aerobic tasks (e.g., cycling or rowing), together with participants’ lack of confidence and fear of injury/pain when performing the prescribed exercises, suggesting the need for active supervision during this type of interventions [63]. This fact can be somewhat confirmed by the results obtained in the only structured and supervised HBE intervention found in this review, which showed significant changes in strength levels in the intervention group compared to the control group [38]. HBE interventions, especially the structured supervised/facilitated ones, might be a good alternative for the most fearful or resistant patients.

This review shows consistent improvements in self-reported PA levels, but studies using objective measures did not confirm these findings. These results align with other studies supporting a pattern of frequent overestimation of PA levels when derived from self-reported instruments, especially aerobic activities, which is also present in cancer survivor populations [64, 65]. This highlights that objectively measured PA should be the preferred method in any population for its precision whenever possible [66].

This review consistently showed high levels of program adherence across different types of HBE programs and treatment phases. This finding is in line with other reviews [17, 20, 67]. The high levels of adherence to the program might be explained by the convenience and affordability of these programs since they do not require any travel or special equipment to be performed [18, 19]. Increasing adherence levels to PA programs and promoting active lifestyles is essential to achieve psychological and physiological health benefits [68]. In contrast, a lower tendency to comply with exercise prescription targets seems to exist. Adherence to the prescribed volume was higher in aerobic training interventions, and intensity targets were better achieved when a moderate rather than vigorous intensity was prescribed. These differences can be related to participants’ exercise history and experienced treatment side effects (e.g., fatigue, pain, discomfort) [69, 70].

Also, it is unclear if HBE interventions effectively promote long-term PA and exercise changes in participants, since most studies did not include follow-up assessments. Of the five studies that did so, only two reported significant increases in PA levels though not significantly different when compared to usual care groups [43, 56]. According to Ki-Yong et al. [70], breast cancer survivors undergoing chemotherapy were more active during the follow-up period of a combined exercise intervention than at baseline, but still showed a decrease in their active behaviors post-intervention. Further research, including longer follow-ups and more assessment timepoints, is necessary to verify if and which types of HBE programs effectively promote long-lasting lifestyle changes in breast cancer survivors.

The information provided by the included studies does not allow us to explore whether the effectiveness of HBE interventions varies according to different primary treatments (i.e., chemotherapy, radiotherapy, and other therapies). This finding aligns with a systematic review [71] that analyzed the effects of exercise interventions during different primary treatments, but could not retrieve conclusions regarding the efficacy of exercise in participants undergoing different treatment regimens due to the limited number of studies. Still, another systematic review [72], investigating the impact of different exercise modes on fatigue during adjuvant chemotherapy and radiotherapy in breast cancer samples found that exercise appears to be effective in reducing fatigue in breast cancer survivors during adjuvant treatments, especially during chemotherapy. This finding suggests that exercise may have a different impact (at least) on some outcomes, depending on the primary treatment regimen. More research exploring the effect of HBE programs on breast cancer survivors undergoing different primary treatment regimens, as well as other types of therapies, is recommended.

HBE interventions were conducted evenly during treatment and post-treatment, but no RCT studies focused on the pre-treatment phase. Nevertheless, prior feasibility studies seem to reveal promising results. A single-arm mixed-methods study [73], investigating the feasibility and acceptability of a tailored HBE prehabilitation intervention before breast cancer surgery, found acceptable completion rates (62%), good adherence to exercise prescription (76%), and clinically significant changes in the 6-minute walk test. Additionally, participants reported having experienced benefits from participating in the program and no exercise-related side effects. Another feasibility study [74] involving breast cancer survivors undergoing hormonal therapy during COVID-19, who performed a 16-week HBE program, reported significant improvements in strength for the upper and lower limbs. Although these studies suggest that an HBE program may be feasible to improve functional performance in the pre-habilitation phase and potentially accelerate recovery, as supervised exercise [73–79], full RCTs are required to confirm these findings.

Almost all the studies in this review conducted aerobic or resistance training interventions alone, not following the PA prescription guidelines for cancer survivors that recommend developing multicomponent exercise programs. Also, these interventions are not entirely aligned with the recommended PA doses for cancer survivors, namely the 150 min or more of at least moderate-intensity PA per week, plus two sessions of muscular resistance training and daily muscle stretching [76, 80, 81]. Stout et al. [80] systematic review also recommends exercising the upper limbs post-surgery in breast cancer survivors. Although aerobic exercise is essential to improve cancer-related fatigue and aerobic capacity, it seems insufficient to enhance upper body strength [78, 79, 82, 83]. The present findings corroborate this idea, given that consistent improvements in aerobic capacity but not in the strength levels of breast cancer survivors were reported. Therefore, this review highlights the relevance of integrating a well-structured resistance training component as part of an HBE program, additionally reinforced by the detrimental effect of cancer treatments on muscle mass and strength [84, 85].

Adverse effects related to exercise were found mostly during treatments but not in the post-treatment phase. However, studies reporting adverse effects referred to unsupervised HBE interventions, again suggesting the need for professional supervision during the treatment phase. Future studies should explore the effects of supervised HBE interventions during treatments to better understand their effectiveness and safety compared to unsupervised ones.

The majority of HBE facilitated interventions used phone calls as a monitoring method. Prior systematic reviews also showed a predominance of phone-delivered PA interventions to breast cancer survivors and a lack of other means of remote delivery methods [86, 87]. However, other delivery methods exist (e.g., wearable devices, websites, email, smartphone apps, and social networking-based) and have been reported as potentially positive to improve PA levels [88–90]. Exploring different delivery methods to tailor the interventions to cancer survivors’ preferences might be interesting, as it can facilitate adopting and maintaining an active lifestyle [91, 92].

### Limitations

To the authors’ knowledge, this is the first systematic review synthesizing the evidence of HBE interventions’ effects on breast cancer survivors’ functional performance and PA levels and their adherence rates, organized by type of HBE intervention and treatment phase.

This review only included RCTs since they are considered the best available evidence to integrate a systematic review for their rigorous design and potential to provide high-quality evidence (REF). Also, a significant strength of this review relates to the inclusion of RCTs that comprised an exercise-only component instead of combined approaches (nutrition, behavioral counseling, among others), allowing the investigation of the unique effects of this component on the outcomes of interest. Still, this review is not without limitations.

Most studies included in this review were conducted in English-speaking countries, which may limit the generalization of the findings to non-English-speaking populations and may not fully capture cultural variations in perceptions of HBE interventions.

This review has focused only on breast cancer (the most studied cancer type), and results cannot be generalized to other cancer types. Most of the studies included in this review (i.e., 79%) presented poor methodological quality, which can lead to biased interpretations and conclusions, thus demanding further research confirming these findings. Also, we only focused on three outcomes of interest (functional performance, PA levels, and program adherence). HBE interventions’ impact on other relevant results (e.g., physiological parameters, quality of life) must be investigated. Furthermore, the high heterogeneity in interventions’ length, exercise modes and doses, type of supervision and control of participants’ effectively performed exercise, and types of treatment being received, among other factors, limit the retrieval of firm conclusions about intervention effectiveness by HBE type and treatment phase. Finally, some types of HBE interventions, namely structured and supervised HBE interventions and the pre-treatment phase, remain scarcely studied, requiring further exploration.

### Clinical implications

Our findings suggest that HBE interventions can be considered safe, especially in a post-treatment phase; effective in raising adherence to PA and promoting a more active lifestyle among breast cancer survivors; and consequently, able to improve cancer survivors’ functional performance and counteract some of the side effects of cancer therapies. The findings from this review give an initial contribution to inform exercise and healthcare professionals on the most appropriate type of HBE programs to be delivered, according to breast cancer survivors’ treatment phase, although additional research is needed. Moreover, along with Denton et al. [16], this review might help researchers classify and describe HBE interventions more accurately in future articles. Upcoming reviews, using Denton’s classification and categorization method, would do well to synthesize the evidence on different cancer populations, compare the effects of HBE interventions with other programs (e.g., in-person or community programs), and analyze their impact on other outcomes, such as fatigue, quality of life, body composition and biomarkers.

## Conclusion

HBE interventions are a feasible strategy to enhance functional performance and raise PA levels in breast cancer survivors, showing high adherence rates. Also, these interventions allow health and exercise professionals to reach people in more remote areas (e.g., rural areas), allowing them to exercise safely and be more active, especially by receiving a prescribed program with supervision/facilitation from a qualified exercise professional, and therefore, to be able to benefit from exercise favorable outcomes. Still, further studies are needed to understand what type of HBE intervention is more suitable for each phase of the survivorship continuum.

## Electronic supplementary material

Below is the link to the electronic supplementary material.

Supplementary Material 1

## Data Availability

Anonymized trial data will be available from the corresponding author upon reasonable request for non-commercial research purposes.
