# Supplementary material for: Home-based exercise interventions’ impact on breast cancer survivors’ functional performance: a systematic review
Source: J Cancer Surviv. 2024 Feb 15;19(4):1222–35. doi: 10.1007/s11764-024-01545-y (PMC12283810; doi:10.1007/s11764-024-01545-y)
Supplement: Supplementary file 1 — Supplementary Material 1 [file 11764_2024_1545_MOESM1_ESM.docx]

**Supplementary Table 1**  Quality appraisal of selected studies using Effective Public Health Practice Project

| Study | Design | Blinding | Selection Bias | Dropouts | Confounders | Data collection | Data analysis | Report | Global rating |
| --- | --- | --- | --- | --- | --- | --- | --- | --- | --- |
| Baruth et al. (2015) | Strong | Weak | Weak | Moderate | Moderate | Strong | Strong | Strong | Weak |
| Cadmus et al. (2009) | Strong | Weak | Weak | Moderate | Moderate | Strong | Strong | Strong | Weak |
| Cornette et al. (2015) | Strong | Weak | Weak | Moderate | Moderate | N/A^1^ | Strong | Strong | Weak |
| Dong et al. (2016) | Strong | Moderate | Weak | Strong | Moderate | N/A | Strong | Strong | Moderate |
| Eakin et al. (2012) | Strong | Moderate | Weak | Strong | Moderate | Strong | Strong | Strong | Moderate |
| Gokal et al. (2016) | Strong | Weak | Weak | Strong | Moderate | N/A | Strong | Strong | Weak |
| Hartman et al. (2018) | Strong | Weak | Weak | Strong | Moderate | Strong | Strong | Strong | Weak |
| Huang et al. (2019) | Strong | Moderate | Weak | Moderate | Moderate | Strong | Strong | Strong | Moderate |
| Husebø et al. (2014) | Strong | Weak | Weak | Moderate | Moderate | Strong | Strong | Strong | Weak |
| Kilgour et al. (2008) | Strong | Moderate | Weak | Strong | Moderate | Strong | Strong | Strong | Moderate |
| Lahart et al. (2016) | Strong | Weak | Weak | Strong | Moderate | Strong | Strong | Strong | Weak |
| Lahart et al. (2018) | Strong | Weak | Weak | Strong | Moderate | Strong | Strong | Strong | Weak |
| Matthews et al. (2007) | Strong | Moderate | Weak | Strong | Moderate | Strong | Strong | Strong | Weak |
| Mcneil et al. (2019) | Strong | Moderate | Weak | Strong | Strong | N/A | Strong | Strong | Weak |
| Mock et al. (2001) | Strong | Weak | Weak | Moderate | Strong | Strong | Strong | Strong | Weak |
| Mock et al. (2005) | Strong | Weak | Weak | Moderate | Moderate | Strong | Strong | Strong | Weak |
| Ochi et al. (2022) | Strong | Moderate | Weak | Strong | Moderate | N/A | Strong | Strong | Moderate |
| Pinto et al. (2005) | Strong | Weak | Weak | Strong | Strong | N/A | Strong | Strong | Weak |
| Pinto et al. (2008) | Strong | Weak | Weak | Strong | Strong | Strong | Strong | Strong | Weak |
| Schwartz et al. (2007) | Strong | Weak | Weak | Strong | Moderate | Strong | Strong | Strong | Weak |
| Sturgeon et al. (2022) | Strong | Weak | Weak | Moderate | Moderate | Strong | Strong | Strong | Weak |
| Wang et al. (2011) | Strong | Weak | Weak | Moderate | Moderate | Strong | Strong | Strong | Weak |
| Yang et al. (2011) | Strong | Weak | Weak | Strong | Moderate | Strong | Strong | Strong | Weak |
| Yuen & Sword (2007) | Strong | Weak | Weak | Strong | Moderate | Strong | Strong | Strong | Weak |
| Farajivafa et al. (2023) | Strong | Weak | Weak | Moderate | Strong | N/A | Strong | Strong | Weak |
| Mavropalias et al. (2023) | Strong | Weak | Weak | Strong | Strong | N/A | Strong | Strong | Weak |
| Abbreviations: ^1^N/A, not applicable; Absent information regarding validity/ reliability but authors provide adequate information to find information about it | | | | | | | | | |

**Supplementary Table 2** Study and outcome details by type of home-based exercise intervention and treatment phase.

| Studies | *Treatment stage* | Sample | Mean Age (Years) | HBE program | Duration | Intervention details | Delivery method | *A*ssessment details | Results |
| --- | --- | --- | --- | --- | --- | --- | --- | --- | --- |
| Structured/ Facilitated HBE intervention during treatment phase | | | | | | | | | |
| Wang et al. (2011), Taiwan | *Post-surgery* | 72 women,  Intervention = 35 Usual care  = 37 | Intervention  = 48.4 yrs Usual care  = 52.3 yrs | Walking program based on Bandura's Self-efficacy Theory | 6 weeks | 30-min, 2-5x/wk 40-60% HR^b^ max or modified RPE^b^ scale 0.5-2 | Weekly phone calls and meetings | 24h pre-surgery (T1), before first CT cycle (T2), 7-10d post-CT (T3), 6-wk (T4) PA: GLTEQ^c^  Aerobic capacity: 6MWT | Exercise behaviour: ↑intervention vs usual care; GLTEQ: ↑ intervention vs usual care  6MWT: ↑intervention vs usual care (T3, T4) |
| Sturgeon et al. (2022), USA | *Neoadjuvant therapy* | 15 women, *during*  Intervention = 7 Usual care  = 8 | Intervention = 51.5 yrs Usual care = 47.0 yrs | Aerobic training | 16-24 weeks | Aerobic DVDs and informational binder 60-75min, 3x/wk,  50-75% baseline VO_2_ max | Weekly phone calls | Baseline (T0), midpoint CT (T1); end of intervention (T2)  Adherence: HR monitors  PA: GLTEQ Aerobic capacity: modified Bruce protocol (VO_2_ max) | Adherence: 87.6%  GLTEQ: ↑intervention vs usual care  VO_2_ max: ↓1.7% Intervention vs ↓24.5% usual care;↑intervention vs usual care |
| Husebø et al. (2014), Norway | *Adjuvant treatment* | *67 women intervention* = 33  usual care  = 34 | Intervention  = 50.8 yrs  Usual care  = 53.6 yrs | Combined aerobic and resistance training | 6 months | Aerobic training: 30-min/d moderate brisk walk  Resistance training: elastic band exercises, 3x/wk | Fortnight phone calls | Baseline (T0), end of CT (T1), follow-up (T2)  Adherence: Exercise diary  PA ^b^: IPAQ^c^ short form  Aerobic capacity: 6MWT^c^ | Adherence: 77.6%; 17% to walking prescription  PA: 58% achieved 150-min/wk; ↔T1, ↑T2; ↔intervention vs usual care  Exercise recommendations: higher tendency to meet from the intervention group  6MWT: ↔T1, ↑T2; ↔ intervention vs usual care |
| Eakin et al. (2012), Australia | *Adjuvant treatment* | 143 women Intervention = 73  Usual care  = 70 | Intervention  = 51.7 yrs  Usual care  = 54.0 yrs | Combined aerobic and resistance training | 8 months | Exercise phone sessions supported by a workbook  Aerobic training: 45 min brisk walk, 4d/wk  Resistance training: 2 sessions/wk, 6-10 exercises, 1-4 sets, 6-12 reps | Phone calls (2-mon weekly, 2-mon fortnightly, 4-mon monthly) | Baseline (T0), 6-mon (T1), and 12-mon post-surgery (T2)  Adherence: Presence registration (phone sessions)  PA: Active Australia Survey  Self-reported functioning: DASH^c^ | Retention: 97% (T1), 96% (T2)  PA: ↔intervention vs usual care  DASH (upper body function): ↑intervention vs usual care |
| (Continue on next page) | | | | | | | | | |

**Supplementary Table 2** Study and outcome details by type of home-based exercise intervention and treatment phase (continued).

| Studies | *Treatment stage* | Sample | Mena Age (Years) | HBE program | Duration | Intervention details | Delivery method | *A*ssessment details | Results |
| --- | --- | --- | --- | --- | --- | --- | --- | --- | --- |
| Structured/ Facilitated HBE intervention during treatment phase | | | | | | | | | |
| Cornette et al. (2015), France | *Neoadjuvant or adjuvant treatment* | 42 women  Intervention  = 20  Usual care  = 22 | Intervention = 52 yrs  Usual care  = 49 yrs | Combined aerobic and resistance training | 27 weeks | Aerobic training: 2 sessions/wk, 20-40 min cycle ergometer or outdoor walk  Resistance training: 1 session/wk, 2 sets, 8-12 reps, w/ elastic bands | Weekly phone calls | Measurements: baseline (T0), end of CT (T1) and 27-wk (T2)  Adherence: exercise diaries, HR monitor  Aerobic capacity: CPET^c^ (VO_2peak_); 6MWT  Strength: isometric bench test | Adherence: 88%  VO_2peak_: ↑ intervention (T1, T2), ↓ usual care,  ↔ intervention vs usual care  Pmax^c^: ↑intervention, ↓usual care (T1, T2),  ↑intervention vs usual care (T1)  Pmax at threshold: ↑intervention (T2), ↑intervention vs usual care (T1)  6MWT: ↑intervention (T1, T2); ↔intervention vs usual care  Strength test: ↔intervention; ↔intervention vs usual care |
| Huang et al. (2019), Taiwan | *Adjuvant treatment* | 159 women  Intervention = 81  Usual care = 78 | Intervention = 48.3 yrs  Usual care = 48.3 yrs | Walking program | 12 weeks | 3 sessions/wk, 15-40 min, 30-70% HR reserve | Weekly phone calls | Adherence: researcher developed weekly exercise logs and HR monitor | Adherence: 80% (prescribed volume = 56.8%,  intensity = 58%) |
| Mock et al. (2001),  USA | *Adjuvant treatment* | 50 women  Low walkers = 22  High walkers = 28  RT ^a^ = 64%  CT ^a^ = 36% | Low walkers = 47.4 yrs  High walkers = 48.6 yrs | Usual care + walking program | 6 weeks RT or 3-6 months CT | 5-6 sessions/wk, 10-30 min | Fortnightly phone calls or clinic visitations | Baseline (T0), mid-treatment (T1), end of treatment (T2)  PA: exercise diaries  Aerobic capacity: 12MWT^c^  Self-reported functioning: MOS-SF36^c^ | Participants crossover: 50% of usual care was active, and 1/3 of intervention was not  PA: ↑high walkers, ↓low walkers, ↔high vs low walkers  12MWT: ↑high walkers, ↔low walkers, ↑high vs low walkers  MOS-SF36: ↓high and low walkers, but more in low walkers |
| (Continue on next page) | | | | | | | | | |

**Supplementary Table 2**  Study and outcome details by type of home-based exercise intervention and treatment phase (continued).

| Studies | *Treatment stage* | Sample | Mena Age (Years) | HBE program | Duration | Intervention details | Delivery method | *A*ssessment details | Results |
| --- | --- | --- | --- | --- | --- | --- | --- | --- | --- |
| Structured/ Facilitated HBE intervention during treatment phase | | | | | | | | | |
| Mock et al. (2005),  USA | *Adjuvant treatment* | 108 women  Intervention = 54  Usual care  = 51  RT= 68%  CT = 42% | Intervention = 51.3 yrs  Usual Care = 51.6 yrs | Walking program | 6 weeks RT or 3-6 months CT | 5-6 sessions/wk, 15-30 min, 50-70% HR max | Fortnight phone calls | Before RT or CT (T0); 6-wk RT or last cycle of CT (T1)  Adherence: weekly diaries  PA: Diaries and PA questionnaire  Aerobic capacity: 12MWT  Self-reported functioning: MOS-SF36 | Adherence: 72% (75% CT, 72% RT)  Participants crossover: 39% of usual care walked >45 min/wk, and 28% of intervention did not  PA: ↑High vs Low exercisers  12MWT: ↑High exercisers; ↓Low exercisers, ↑High vs Low exercisers  MOS-SF36:↑High exercisers, ↓Low exercisers, ↑High vs Low exercisers |
| Schwartz et al. (2007), USA | *Adjuvant treatment* | 66 women  Aerobic intervention = 22  Resistance intervention = 21  Usual care = 23 | Aerobic intervention = 48.32 yrs  Resistance intervention = 50.1 yrs  Usual care = 46.26 yrs | Aerobic training or resistance training | 6 months | Aerobic training: 4d/wk, 15-30 min, moderate-intensity self-chosen aerobic activity  Resistance training: 4d/wk w/ resistance bands  2 alternating groups of 8 exercises, 2 sets of 8-10 reps | Fortnight (months 1 and 2) and monthly (months 3-6) phone calls | Baseline (T0), 6-months (T1)  Aerobic capacity: 12MWT  Strength: 1-repetition maximum | 12MWT: ↑Aerobic vs resistance intervention and usual care Strength: ↑Aerobic vs resistance intervention and usual care on seated row and leg extension |
| Yang et al. (2011), Taiwan | *Adjuvant treatment* | 40 women  Intervention = 19  Usual care = 21 | Intervention = 50.79 yrs  Usual care = 52.71 | Walking program | 12 weeks | 3d/wk, 5-min warm-up, 30-min brisk walk, 60-80% HR max, 5-min cool down | Weekly phone calls | Baseline (T0), 6-wk follow-up (T1), end of intervention (T2)  Adherence: HR monitor  PA: 7-Day PA Recall | Adherence: 77%  ↔ energy expenditure time by group (T0 vs T1, T2)  PA levels: ↑group by time; ↑ intervention vs usual care |
| (Continue on next page) | | | | | | | | | |

**Supplementary Table 2**  Study and outcome details by type of home-based exercise intervention and treatment phase (continued).

| Studies | *Treatment stage* | Sample | Mena Age (Years) | HBE program | Duration | Intervention details | Delivery method | *A*ssessment details | Results |
| --- | --- | --- | --- | --- | --- | --- | --- | --- | --- |
| Structured/ Facilitated HBE intervention during treatment phase | | | | | | | | | |
| Mavropalias et al. (2023), Australia | *Adjuvant*  *treatment* | 106 women  Intervention = 51  Usual Care  = 55 | Intervention = 51 yrs  Usual care  = 53 yrs | Combined resistance and aerobic training | 12 weeks | 30-min/d, 5d/wk of moderate PA or 20-min/d, 3d/wk of vigorous PA)  8-10 resistance exercises, 8-12 reps per exercise, 2-3d/wk | Regular oncologist reinforcement  1-h presential consultation  30-min phone consultations every 2wk | Baseline (prior to RT); Post-RT (6-wk); Post-exercise (12-wk); Follow-up (6 and 12 months)  GLTEQ | Moderate to vigorous PA: ↑ intervention (6 wk, 6 and 12 months)  ↔ Intervention vs Usual Care |
| Structured/ Facilitated HBE intervention post-treatment phase | | | | | | | | | |
| Baruth et al. (2015),  USA | *≤ 12-months* | 32 women, post-menopausal  Intervention = 20  Usual care  = 12 | Intervention = 54.9 yrs  Usual care = 57.4 yrs | Walking program | 12 weeks | 3-5d/wk, 20-40min session, moderate to vigorous intensity | Phone calls (based on social cognitive theory) in weeks 1,2,4,7 and 10 | Baseline (T0), end of study (T1)  Adherence: activity logs  PA: CHAMPS^c^ | Adherence: 86.2%  Energy expenditure from walking: ↑intervention vs usual care |
| Mcneil et al. (2019), Canada | *Hormone therapy* | 45 women  Low-intensity PA = 15  High-intensity PA = 15  Usual care = 15 | Low-intensity PA  = 58 yrs  High-intensity PA = 58 yrs  Usual care = 60 yrs | Any aerobic activity | 12 weeks | Low-intensity PA: 300-min/wk at 40-59% HR reserve  High-intensity PA: 150-min/wk at 60-80% HR reserve  Any aerobic activity, including household | Phone calls or  email | Baseline (T0), end of intervention (T1), 24-wk follow-up (T2)  PA: accelerometer, activity tracker and diary  Aerobic capacity: Balke treadmill test ((VO_2peak_), Pollocks formula | PA in target HR: ↑Low- vs High-intensity PA group  Accelerometer measured PA: ↑Low intensity vs usual care; ↔High-intensity vs usual care  VO_2peak_: ↑Low-vs High-intensity PA (T0, T1), ↑Low- and High-intensity PA vs Usual care (T1), ↔Low- and High-intensity vs Usual care (T2) |
| (Continue on next page) | | | | | | | | | |

**Supplementary Table 2**  Study and outcome details by type of home-based exercise intervention and treatment phase (continued).

| Studies | *Treatment stage* | Sample | Mean Age (Years) | HBE program | Duration | Intervention details | Delivery method | *A*ssessment details | Results |
| --- | --- | --- | --- | --- | --- | --- | --- | --- | --- |
| Structured/ Facilitated HBE intervention post-treatment phase | | | | | | | | | |
| Matthews et al. (2007), USA | *≤ 12-months* | 36 women, post-menopausal  Intervention = 22  Usual Care = 14 | Intervention = 51.3 yrs  Usual care = 56.9 yrs | Walking program | 12 weeks | 3-5 sessions/wk, 20-40 min, moderate intensity | Phone calls, week 1,2,4,7 and 10 | Baseline (T0), end of study (T1)  Adherence: walking logs calculations  PA: CHAMPS, accelerometer | Adherence: 96.6% (6-wk), 74.9% (12-wk)  PA: ↑intervention, ↑intervention vs usual care  Self-reported walking: ↑intervention vs usual care  Accelerometer: ↑intervention, group-by-time interactions in activity count and steps; ↔intervention vs usual care |
| Ochi et al. 2022,  Japan | *Not reported* | 82 women  Intervention = 21  Usual Care =23 | Age range = 20-59 yrs | High-intensity interval training | 12 weeks | Body weight exercises, 3d/wk, 10-min session, guided through app | App and personalised email | Baseline (T0), end of intervention (T1)  Adherence: smartwatch and app  Aerobic capacity: cycle ergometer test (VO_2peak_), 6MWT^c^  Strength: 1 Repetition maximum Leg press test, grip strength, chair stand test | Adherence: 86%  VO_2peak_: ↑intervention vs usual care  6MWT: ↔intervention vs usual care  Leg press: ↑intervention vs usual care;  Grip strength: ↔intervention vs usual care  Chair stand test: ↔intervention vs usual care |
| Pinto et al. (2005),  USA | *≤ 5 yrs* | 82 women  Intervention = 39  Usual care = 43 | Intervention = 53.4 yrs  Usual care = 52.86 yrs | Walking program | 12 weeks | Based on the transtheoretical model  2-5d/wk, 10-30 min, at 55-65% HR max | Weekly phone calls | Baseline (T0), end of intervention (T1)  PA: 7-Day PA Recall, accelerometer; pedometer  Aerobic capacity: Rockport  1-mile walk | Total min and moderate intensity: ↑intervention vs usual care  7-Day PA Recall: ↑intervention vs usual care  Accelerometer: ↔intervention vs usual care  Pedometer steps: ↑intervention vs usual care  1-mile walk: ↑intervention vs usual care |
| (Continue on next page) | | | | | | | | | |

**Supplementary Table 2**  Study and outcome details by type of home-based exercise intervention and treatment phase (continued).

| Studies | *Treatment stage* | Sample | Mean age (years) | HBE program | Duration | Intervention details | Delivery method | *A*ssessment details | Results |
| --- | --- | --- | --- | --- | --- | --- | --- | --- | --- |
| Structured/ Facilitated HBE intervention post-treatment phase | | | | | | | | | |
| Pinto et al. (2008),  USA | *≤ 5 yrs* | 86 women  Intervention = 43  Usual care = 43 | Intervention = 53.42 yrs  Usual care = 52.86 yrs | Walking program | 12 weeks | 2-5d/wk, 10-30 min, moderate intensity | Weekly phone calls and mailed tip sheets | Baseline (T0), end of intervention (T1), 6-month follow-up (T3), 9-month follow-up (T4)  PA: 7-Day PA Recall  Aerobic capacity: Rockport  1-mile walk | 7-Day PA Recall: group by time interaction, ↑intervention vs usual care (T1, T4)  1-mile walk: ↑intervention vs usual care (T1, T4) |
| Yuen & Sword (2007),  USA | *Not reported* | 22 women  Aerobic group = 7  Resistance group =8  Usual care =7 | Aerobic group  = 55.0yrs  Resistance group = 53.7 yrs  Usual care = 53.1 yrs | Aerobic or resistance training intervention | 12 weeks | Aerobic training: 3d/wk, 20-40 min moderate walking or other activities  Resistance training: 3 non-consecutive d/wk, 8 exercises w/ dumbbell, 1-3 circuits, 8-12 reps | Weekly phone calls (first 3-wks) | Baseline (T0), end of intervention (T1)  Adherence: exercise logs  Aerobic capacity: 6MWT | Adherence: 72.7% (Aerobic group),  79.8% (Strength group)  6MWT: ↑Strength, but not aerobic group |
| Farajivafa et al. (2023), Iran | *≤ 1 year post-treatment* | 89 women  Intervention = 44  Usual Care = 45 | Intervention = 45.05 yrs  Usual Care = 46.6 yrs | Walking, balance exercises, and stretches | 12-weeks | From 15-min/wk, 2d/wk to 50-60 min/session, ≥ 3d/wk or 30-40 min/session, 5d/wk | Weekly phone call | Baseline, Post-intervention (12-wk)  Adherence: exercise logs  Aerobic capacity: single-stage submaximal treadmill walking test  Heartrate monitor | Adherence: 90%  Aerobic capacity: VO_2peak_ ↑ Intervention vs Usual Care |
| (Continue on next page) | | | | | | | | | |

**Supplementary Table 2**  Study and outcome details by type of home-based exercise interventions and treatment phase (continued).

| Studies | *Treatment stage* | Sample | Mean Age (Years) | HBE program | Duration | Intervention details | Delivery method | *A*ssessment details | Results |
| --- | --- | --- | --- | --- | --- | --- | --- | --- | --- |
| *Structured/ Unsupervised HBE intervention post-treatment* | | | | | | | | | |
| Kilgour et al. (2008), Canada | *Post-surgery (mastectomy and sentinel node dissection)* | 27 women  Intervention = 16  Usual care = 11 | Intervention = 50.6 yrs  Usual care = 49.1 yrs | Shoulder range of motion and flexibility | 11 days | Exercise video program,  Phase I: 3 sets/d, 5-7 min/set  Phase II: 2 sets/day, 10-15 min/set | Not applicable | 3d post-surgery (T0), post-intervention (T1)  Adherence: diary logbook  Range of motion: standard goniometric techniques  Strength: muscle testing techniques, grip strength (dynamometry) | Adherence: 50%  Shoulder flexion and abduction: range of motion, ↑intervention vs usual care, ↔ strength  Shoulder external rotation: ↔intervention vs usual care in range of motion |
| Gokal et al. (2016),  UK | *Not reported* | 50 women  Intervention = 25  Usual care = 25 | Intervention = 52 yrs  Usual care = 52 yrs | Walking program | 12 weeks | Usual care + self-managed schedule walking, based on the Theory of Planned Behaviour and supported by a booklet  5d/wk, 10-30 min at moderate intensity | Not applicable | Baseline (T0), end of intervention (T1)  Adherence: exercise logbook  PA: GPPA^c^, exercise diary | Adherence: 80%  PA: ↑intervention vs usual care |
| Structured/ Supervised HBE intervention post-treatment phase | | | | | | | | | |
| Dong et al. (2019), China | *4 months – 2 yrs post RT or CT* | 50 women  Intervention = 26 Usual care = 24 | Intervention = 51.3 yrs  Usual care = 56.9 yrs | Combined televideo aerobic and resistance training | 12 weeks | Aerobic training: 4 sessions/wk, completing target number of steps in a certain time  Resistance training: endurance (month 1), strength (month 2), muscle function (month 3) 3 sessions/wk, 30-min (5-min warm-up, 20-min muscle training, 5-min cooldown) | Televideo | Baseline (T0), end of intervention (T1)  Aerobic capacity: VO_2peak_  Strength: sit-to-stand and arm lifting test | VO_2peak_: ↑intervention; ↔ intervention vs usual care  Sit-to-stand and arm lifting test: ↑intervention; ↑ intervention vs usual care |
| (Continue on next page) | | | | | | | | | |

**Supplementary Table 2**  Study and outcome details by type of home-based exercise interventions and treatment phase (continued)

| Studies | *Treatment stage* | Sample | Mean Age (Years) | HBE program | Duration | Intervention details | Delivery method | *A*ssessment details | Results |
| --- | --- | --- | --- | --- | --- | --- | --- | --- | --- |
| Unstructured/ Supervised HBE intervention during treatment phase | | | | | | | | | |
| Cadmus et al. (2009), USA | *Adjuvant treatment* | 50 women  Intervention = 25  Usual care  = 25 | Intervention = 54.5 yrs  Usual care = 54.0 yrs | PA promotion  Aerobic training | 6 months | Program based on planned behaviour theory and transtheoretical model + educational book and binder with information handout + HR monitor  5d/wk, 30min PA at 60-80% of predicted HR max of aerobic activities | Weekly phone calls | Baseline (T0), end of intervention (T1)  Adherence: 7-day PA log;  7-day pedometer log | Adherence: 90% |
| *Unstructured/ Facilitated HBE intervention post-treatment phase* | | | | | | | | | |
| Hartman et al. (2018), USA | *< 5 yrs* | 87 women  Intervention = 43  Usual care = 44 | Intervention = 58.2 yrs  Usual care = 56.2 yrs | PA promotion intervention  Aerobic exercise | 12 weeks | Motivational interview to set goals and strategies to achieve 150 min of moderate to vigorous PA/wk | Phone calls and emails | Baseline (T0), end of intervention (T1)  PA: accelerometer | Min/day total and MVPA: ↑intervention vs usual care  Target MVPA: ↑intervention vs usual care |
| Lahart et al. (2016),  UK | *≤ 2 yrs after all treatment but hormone therapy* | 80 women  Intervention = 40  Usual care = 40 | Intervention = 52.4 yrs  Usual care = 54.7 yrs | Active lifestyle promotion  Recommended aerobic activities | 6 months | PA promotion w/ booklet, DVD  Target: 3-7d/wk, 30-min of the preferred type of PA, at moderate intensity | In-person consultations, phone calls,  and mailed PA encouragement leaflets | Baseline (T0), end of intervention (T1)  PA: IPAQ long-form | Total PA: ↑intervention vs usual care  Leisure and vigorous PA: ↑intervention vs usual care |
| (Continue on next page) | | | | | | | | | |

**Supplementary Table 2**  Study and outcome details by type of home-based exercise interventions and treatment phase (continued)

| Studies | *Treatment stage* | Sample | Mean Age (Years) | HBE program | Duration | Intervention details | Delivery method | *A*ssessment details | Results |
| --- | --- | --- | --- | --- | --- | --- | --- | --- | --- |
| *Unstructured/ Facilitated HBE intervention post-treatment phase* | | | | | | | | | |
| Lahart et al. (2018),  UK | *≤ 2 yrs after all treatment but hormone therapy* | 32 women  Intervention = 16  Usual care = 16 | Intervention = 52.5 yrs  Usual care = 52.0 yrs | Active lifestyle promotion  Recommended aerobic activities | 6 months | PA promotion w/ booklet, DVD  Target: 3-7d/wk, 30-min of the preferred type of PA, at moderate intensity | In-person consultations, phone calls,  and mailed PA encouragement leaflets | Baseline (T0), end of intervention (T1)  PA: IPAQ long-form  Aerobic capacity: Bruce protocol on treadmill (VO_2peak_) | VO_2peak_: achieved in almost all participants at baseline and post-intervention  Magnitude-based inference analysis: potential benefit in relative (85%) and absolute VO_2peak_ (82%), favouring intervention;  Potential benefit for total (91%) and moderate PA (82%)  ↔ vigorous PA; VO_2peak_ |
| Abbreviations: ^a^ HBE, Home-based exercise; CT, chemotherapy; RT, radiotherapy; ^b^ HR, heart rate; RPE, Rating Perceived Exertion, PA, physical activity  ^c^ IPAQ, International Physical Activity Questionnaire; GLTEQ, Goldin Leisure Time Exercise Questionnaire; 6MWT, 6-minute walk test; DASH, Disability of Arm, Shoulder, and Hand questionnaire; CPET, cardiopulmonary exercise test; 12MWT, 12-minute walk test; MOS-SF36, Medical Outcomes Survey Short-Form; CHAMPS, Community Healthy Activities Models Programs for Seniors; GPPA, General Practice Physical Activity; Pmax, maximum power  ↑ positive effect from the intervention or significant differences between groups; ↔ no effect or no differences between groups; ↓ decreases | | | | | | | | | |
